# Genetic and chemodiversity in native populations of *Schinus terebinthifolia* Raddi along the Brazilian Atlantic forest

**DOI:** 10.1038/s41598-021-00015-0

**Published:** 2021-10-14

**Authors:** Jannaina Velasques, Bruno do Amaral Crispim, Adrielle Ayumi de Vasconcelos, Miklos Maximiliano Bajay, Claudia Andrea Lima Cardoso, Alexeia Barufatti, Maria do Carmo Vieira

**Affiliations:** 1 Programa de Pós-Graduação em Biossistemas, Universidade Federal do Sul da Bahia, Itabuna, Bahia Brazil; 2 Programa de Pós-Graduação em Biodiversidade e Meio Ambiente, Faculdade de Ciências Biológicas e Ambientais, Universidade Federal da Grande Dourados, Dourados, Mato Grosso do Sul Brazil; 3 Departamento de Genética, Evolução, Microbiologia e Imunologia, Instituto de Biologia, Universidade Estadual de Campinas, Campinas, São Paulo Brazil; 4 Departamento de Engenharia de Pesca e Biologia, Universidade do Estado de Santa Catarina, Florianópolis, Santa Catarina Brazil; 5 Programa de Pós Graduação em Recursos Naturais, Universidade Estadual de Mato Grosso do Sul, Dourados, Mato Grosso do Sul Brazil; 6 Programa de Pós-Graduação em Agronomia, Faculdade de Ciências Agrárias, Universidade Federal da Grande Dourados, Dourados, Mato Grosso do Sul Brazil

**Keywords:** Plant sciences, Natural variation in plants, Plant genetics

## Abstract

*Schinus terebinthifolia* is a species native to different ecoregions in the Brazilian Atlantic Forest. The plant is listed on the National Relation of Medicinal Plants and recommended as phytomedicine, however while extractive exploitation prevails as the main route of raw material a significant variation of compounds will be detected. To assure the expansion of productive chain it is important to start by studying population diversity and chemical variations. We used SSR markers for studies of genetic structure among populations from dense ombrophilous forest (ES); the deciduous seasonal forest (SM); the savanna (DOU) and the sandbanks (ITA and MSP), and compared the results to their chemical profiles of essential oil. Genetic structure revealed differences among populations and significant fixation rates. Pairwise studies and Bayesian analysis showed similarities between ITA and SM and between DOU and MSP, proving that the patterns of distribution for the species do not follow the isolation by distance or similarity by environmental conditions. The comparison between PCA of genotypes and chemodiversity reinforces the unique profile for each population despite the environmental similarity observed and genetic analysis. The most divergent genotype and chemical group was found at the ombrophilous forest, strong evidence that we should undertake conservation efforts to prevent losses of biodiversity in that area.

## Introduction

*Schinus terebinthifolia* Raddi (Anacardiaceae) is an aromatic spice commonly known as pink pepper, Brazilian peppertree, faux poivrier, baie rose. The species is native to the Brazilian Atlantic Forest and has considerable ecoplasticity. In Brazil, it can be seen throughout several ecosystems from the sandbanks in south Pernambuco (08° 03′ 14″ S and 34° 52′ 52″ W) to the deciduous seasonal forests in Rio Grande do Sul (30° 01′ 59″ S and 51° 13′ 48″ W). As a small evergreen tree, it grows up to 3–13 m and behaves as a pioneer in degraded areas showing rapid and aggressive development by preventing regeneration of other native species. There’s a historical link attributing the interest in the aromatic and spicy features of its fruits to the spread of the species during Brazilian colonization, followed by recent re-introductions for ornamental purposes. Hence, it’s now dispersed worldwide and colonizing abandoned agricultural areas, degraded forests, coastal ecosystems, wetlands, and riparian zones, which has influenced its ranking among the 100 worst invasive species on the planet^1–5^.

On the other hand, the Brazilian peppertree is well known for its medicinal properties. The leaves and the fruits are widely used in traditional medicine as anti-inflammatory, antipyretic, analgesic and purifying agents^6^. In recent years, a number of pharmacological studies have confirmed its antimicrobial, antioxidant, vasodilatory, and antitumor activities. These studies have encouraged the Brazilian Ministry of Health to include the Brazilian peppertree among the species of therapeutic potential to be distributed by the National Health System (SUS)^7–16^.

Therefore, a series of studies encouraging the propagation of Brazilian peppertree by family farming and its use for reforesting degraded areas increased substantially. Some authors, considering the idea of the occurrence of chemotypes, defend the necessity of preventing extractive exploitation and the management of units for conservation of the species^17,18^. However, the Brazilian peppertree has a short generation time, is a highly prolific seed producer, and the seeds are readily consumed and spread by birds^2,19,20^, it is imperative to take into account the global warning about the difficult control of Brazilian peppertree dispersion and the risks it implies to the environment.

Studies of genetic diversity and spatial structure were carried out with distinct populations of Brazilian peppertree^5,18–21^. Nevertheless, there are still many gaps related to the distribution and dispersion of the Brazilian peppertree, mainly because the sample groups in previous works have always been restricted to a few native ecogeographic regions. Essential oil composition may be affected by a variety of factors including soil properties, solar radiation, temperature, altitude, humidity and ontogenetic stage, and physiological stages of fruit maturation^22–24^. The sum of all these factors can potentially affect the bioactivity of a final product. Studies comparing the genetic diversity and chemodiversity of the species across different environmental conditions for growth is crucial for the development of elite lines for traditional medicine and the pharmaceutical industry.

The essential oil of the Brazilian peppertree is already used in the formula of many cosmetics and pharmaceutical products. Although considering the heterogeneity of its composition observed on pharmacological studies, we could not find in the literature studies in comparison of chemodiversity of the species considering environmental conditions of growth. Nevertheless, major constituents often observed are α-pinene, limonene, sabinene, β-pinene, α-copaene, α-phellandrene, germacrene and cis-salvene^8,25–28^, with a great variation among their concentration.

In this study, we compare genetic diversity and chemodiversity among five populations representing distinct Brazilian ecosystems: decidual seasonal forest from Santa Maria—Rio Grande do Sul (Cfa); savanna from Dourados—Mato Grosso do Sul (Cwa); dense ombrophilous forest from São Mateus—Espírito Santo (Aw); sandbanks from Itaparica—Bahia (Af) and from Morro de São Paulo—Bahia (Af). We ask if differences in chemodiversity may be related to ecoregion or genetic differences among these populations.

## Materials and methods

### Plant material and DNA extraction

To access the plant material in this study, the authors followed all Brazilian legal frameworks (Law 13,123/15 and Decree 8772/16) to Genetic Heritage for purpose of scientific research. A former Certificate was emitted by the Genetic Heritage Management Council in the name of Universidade Federal da Grande Dourados and can be accessed in the National System of Genetic Resource Management and Associated Traditional Knowledge (SISGEN nº A9CDAAE). The material collected never left Brazilian territory and all the analysis were made in our laboratory under our legislation and security.

**Figure 1:**
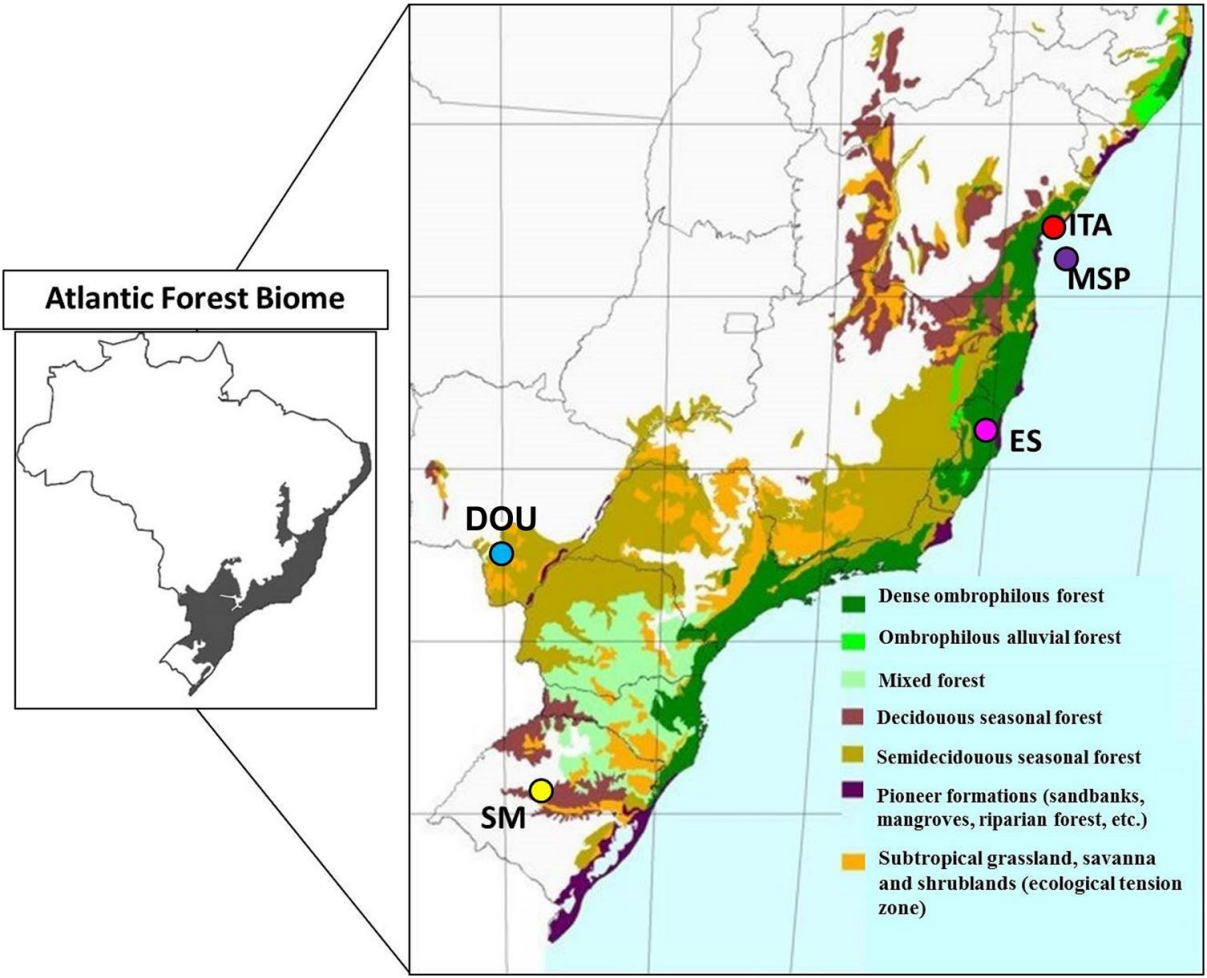
Distribution of *S. terebinthifolia* study population in different Brazilian ecosystems, all of them located in the Atlantic forest biome. Collected in ES, *São Mateus-ES*; SM, *Santa Maria-RS*; ITA, *Vera Cruz–BA*; MSP, *Morro de São Paulo–BA*; DOU, *Dourados–MS*. (Map of the original vegetal physiognomy. Source: INPE, http://mapas.sosma.org.br/).

Fresh young leaves of native accessions were randomly collected to represent ecogeographical groups in Brazil (Table 1, Fig. 1). A voucher specimen of each location was identified by the specialist Dr Zefa Valdivina Pereira and deposited in the herbarium collection of Universidade Federal da Grande Dourados (DDMS 4872, 4873, 4874, 4875 and 4876, respectively for ITA, MSP, ES, SM and DOU).

**Table 1:** Georeferences and climate information of native populations of *S. terebinthifolia* from five Brazilian ecosystems in the Atlantic Forest biome, used for population genetics and chemodiversity studies.

Genotype	Origin	Ecosystem	Latitude (S)	Longitude (W)	Altitude (m)	Climate	PI (mm)	T°C
ES	São Mateus ES	Dense ombrophilous forest	18° 43′ 00″	39° 51′ 31″	38	Aw	1313	24.1
SM	Santa Maria RS	Deciduous seasonal forest	29° 41′ 02″	53° 48′ 25″	151	Cfa	1617	18.8
ITA	Vera Cruz BA	Sandbanks	12° 57′ 32″	38° 36′ 16″	13	Af	1874	25.1
MSP	Morro de São Paulo BA	Sandbanks	13° 23′ 22″	38° 54′ 36″	19	Af	2151	25.3
DOU	Dourados MS	Savanna	22° 13′ 15″	54° 48′ 21″	430	Cw	1400	23.6

BA, *Bahia*; ES, *Espírito Santo*; MS, *Mato Grosso do Sul*; RS, *Rio Grande do Sul*; PI, *Pluviometric index*; Af, Tropical rainforest climate; Aw, Tropical wet and dry climate; Cfa, Subtropical fully humid hot summer; Cwa, Subtropical dry winter hot summer.

After collection, the plant material was sterilized in a 3% NaClO solution for 10 min, then rinsed, dried and stored in liquid nitrogen until DNA extraction. Total genomic DNA was extracted from leaf tissue using the CTAB method^29^.

### Microsatellites

We used a panel of seven microsatellite loci adapted from Williams et al.^30^ (StAAT1, StAAT9, StAAT16, StAAT17, StAAT25, StAAT47, StAAT55) for polymorphisms. PCR were performed using a multiplex system as described in Table 2.

**Table 2:** Polymorphic markers used to genotype native populations of *S. terebinthifolia*. Microsatellite loci, primer sequences (5′–3′), annealing temperature (°C), number of alleles (NA) and accession numbers.

Multiplex	Locus	Primer sequences 5′–3′	T (°C)	NA	Genbank accession nº
M1	*St*AAT25^a^	ATTTGGAAAATAATAATAATAATA	50°	6	AF404285.1
		CGTGCAGAACTTCAATTTTGATTG			
	*St*AAT55^a^	AAGGGGTTAAAAAATAATCAAACT	50°	10	AF404289.1
		TAATAACTATGTTAGGTTAGATAG			
M2	*St*AAT16^b^	AACAGCCCACCATTTTAACAA	50°	7	AF404282.1
		TGGGTAGGTGATGCAGTTCTA			
	*St*AAT47^b^	CCTCTTAAGGAACTTTTTATTATT	50°	4	AF404287.1
		TTGCTTTTCATTTGTTTATCTTAC			
M3	*St*AAT17^a^	TTGGGTTAAATTGGTAGGTGTAAT	50°	7	AF404283.1
		AGGGGAAATGAAATCATCCTTATT			
	*St*AAT9^b^	ATTTGGTGAATAGGAGATGTTTTA	50°	7	AF404280.1
		ATTGAAATGTTTGGTTCATAGATA			
	*St*AAT1^a^	AAGGGTGAGAATCTGAAATTTA	50°	12	AF404279.1
		GGCAAACCCATTAGTGAGTTTA			

^a^Primers redesigned based on total sequences published on Genbank databases, adapted from Williams et al.^30^.

^b^Primers designed according to Williams et al.^30^.

PCR final volume (25 μl) contained: 4.5 µl of ultrapure water, 12.5 µl of PCR master mix, 1.0 µl of each primers, 2U of Taq DNA Polymerase and 2.3 µl of DNA (25–30 ng). The thermocycling program consisted of an initial denaturation at 94 °C for 2 min, followed by 30 denaturation cycles at 94 °C for 30 s, annealing at 50 °C for 30 s and extension at 72 °C for 30 s. A final extension step at 72 °C for 10 min was performed at the end of the 30 cycles.

Amplified products were separated in 6% polyacrylamide gel, prepared on a 34-well sandwich type glass plate. A pre-run of 50 min at 70 W was carried out before applying the PCR samples. The 5 μL aliquot from PCR product was applied and the electrophoresis run lasted for approximately 2 h, with constant 70 W. A 10 bp ladder molecular weight marker was loaded on the side edge of each gel to assign a fragment size to each band. The gels were stained with silver nitrate.

### Data analysis

Parameters of loci diversity were estimated for all microsatellites, in all populations, using the CERVUS 3.0 program^31^. The parameters were as follows: allele frequency was estimated by direct counting, expected (He) and observed heterozygosity (Ho), polymorphic information content (PIC) and Hardy–Weinberg equilibrium (HWE). The diveRsity R package (R Development Core Team 2020)^32^ was used to calculate allelic richness (AR) and estimates of Wright's F statistics (F_IT_, F_IS_ and F_ST_). Confidence intervals were obtained with 10,000 bootstrap replicates. The influence of null alleles was determined in FREENA^33^ by computing the genetic divergence parameter (F_ST_) values using an excluding null alleles (ENA) correction. After accounting for null allele frequencies, loci with frequencies of ≥ 0.2 were considered potentially problematic for the calculations.

The P-value was adjusted using the Bonferroni procedure with the same statistical package. Population structure was evaluated by analysis of molecular variance (AMOVA) using the ARLEQUIN program version 3.5.2.2^34^ to reveal the diversity within localities and among localities. The dendrogram was constructed by cluster analysis using the UPGMA method, based on calculations of Nei's genetic distances and 1000 bootstrap resamplings using poppr R package^35^.

Based on the allelic frequency of the seven microsatellite loci, the individuals were grouped in a given number of populations and probabilistically marked into groups inferred by Bayesian analysis using the Structure program^36^. The tests were performed using an admixture model where the allelic frequencies were correlated. To select the appropriate number of inferred populations, several analyzes were conducted with k (number of populations inferred) ranging from 2 to 6, with 30,000 interactions (burn-in period of 300), with three independent replications for each analysis. The real values of K were inferred from the magnitude of ΔK and given as a function of K, with the aid of the Structure Harvester program^37^ following the model proposed by Evanno et al.^38^ To corroborate the outputs from the Bayesian analysis, we also analyzed the datasets via a discriminant analysis of principal components (DAPC)^39^ using the ADEGENET package^40^. The number of clusters was assessed using the function *find.clusters*, which runs successive rounds of K-means clustering with an increasing number of clusters (K). To select the optimal number of clusters, we chose the lowest associated Bayesian information criterion (BIC) value.

### Chemical analysis of essential oil

We used a Clevenger-type apparatus for the extraction of the essential oil from ripe berries of Brazilian peppertree. A pool of fruits from several individuals representative from each ecogeographic region was weight (300 g) and submitted to 3 h of hydrodistillation. The Fig. 4 shows the moment and quality of fruits harvested (images with participant consent).

The essential oil was stored in sterile microtubules at − 20 °C until further analysis. The samples were prepared in hexane at the concentration 100 µg mL^−1^. The gas chromatograph used was a GC-2010 Plus (Shimadzu, Kyoto, Japan) coupled to a mass spectrometer (GC–MS 2010 Ultra) using a DB-5 column (J and W, Folsom, California, USA) coated with 5% phenyl dimethylpolysiloxane on capillary fused silica (30 m long × 0.25 mm internal diameter × 0.25 μm film thickness). The conditions of analysis were as follows: injection volume 1 µL in split 1:20 mode; heating ramp with initial temperature of 50 °C, reaching 280 °C at a rate of 3 °C min^−1^ and remaining at the final temperature for 10 min; and injector temperature of 280 °C. The temperatures of detector and transfer line were 290 °C. The parameters of mass spectrometry included scanning MS voltage electron impact ionization of 70 eV with *m/z* 40–600 and scanning range of 0.3 s. Compound identifications were performed using the calculated retention index (RI) and the linear alkane (C_7_–C_40_, Sigma Aldrich purity ≥ 90%) standard, along with comparisons of the RI with indexes found in the literature^41^ and also used our interpretation of mass spectra obtained from the samples and compared with the databases (NIST21 and WILEY229). The peak area of each compound was determined by manual integration of each total ion chromatogram (TIC). Then all areas were transformed into relative percentage areas (relative abundance percentual).

### Ethical approval

The access to genetic resources were assured by SISGEN license nº A9CDAAE.

## Results

Population structure analysis revealed differentiation among them of 37.72% (Table 3) and significant fixation index based on F_ST_ (P < 0.001).

**Table 3:** Analysis of Molecular Variance (AMOVA) of *S. terebinthifolia* in five ecoregions of the Atlantic forest biome.

Variance source	DF	Variation (%)	FI
Interpopulation	4	37.72	F_ST_ = 0.377*
Intrapopulation	145	62.68	

*DF* degree of freedom, *FI* fixation index.

*P < 0.001.

The parameters evaluated for genotypic characterization of the populations based on seven microsatellite loci are described in Table 4. The population ES (São Mateus-ES) had the higher allelic richness (4.98), with the inbreeding coefficient being significantly different from zero (0.16), the greatest expected heterozygosity was shown by the population of SM (Santa Maria-RS) (0.53). The DOU population (Dourados-MS) presented the lower allelic richness (2.43); but the lower expected heterozygosity (0.43) was observed in MSP (Morro de São Paulo-BA).

**Table 4:** Genetic diversity parameters of native populations of *S. terebinthifolia* from five ecoregions in the Atlantic Forest biome using microsatellites markers.

Genotype	N	Heterozygosity		AR	F_IS_	Loci in HWE
		Observed	Expected			
ES	30	0.52 ± 0.03	0.64 ± 0.07	4.98	0.16 (0.05 0.28)*	4
SM	31	0.57 ± 0.03	0.53 ± 0.08	4.06	− 0.08 (− 0.19 0.02)	3
ITA	26	0.52 ± 0.04	0.51 ± 0.04	3.70	− 0.04 (− 0.19 0.09)	2
MSP	28	0.39 ± 0.04	0.43 ± 0.07	3.10	0.09 (− 0.01 0.18)	3
DOU	30	0.58 ± 0.03	0.46 ± 0.09	2.43	− 0.24 (− 0.35 − 0.12)*	4

Values between parenthesis correspond to 95% confidence interval calculated using 1000 bootstrap resamples.

N, number of individuals; AR, allelic richness; F_IS_, coefficient of inbreeding; HWE, number of loci showing deviations from Hardy–Weinberg Equilibrium; ES, São Mateus ES; SM, Santa Maria RS; ITA, Itaparica BA; MSP, Morro de São Paulo BA; DOU, Dourados MS.

When analyses were performed considering all populations, the seven microsatellite loci showed Hardy–Weinberg equilibrium. However, when the populations were evaluated separately, the loci in HWE were: ES (*St*AAT01, *St*AAT09, *St*AAT17, *St*AAT55); SM (*St*AAT01, *St*AAT16, *St*AAT55); ITA (*St*AAT01, *St*AAT09); MSP (*St*AAT01, *St*AAT09, *St*AAT17) and DOU (*St*AAT01, *St*AAT16, *St*AAT17, *St*AAT55). After null allele correction (ENA), overall F_ST_ changed only slightly (from 0.377 to 0.369). Pairwise F_ST_ values were highest for DOU, followed by MSP while FST was the lowest between ITA and SM (Table 5).

**Table 5:** Pairwise F_ST_ among native populations of *Schinus terebinthifolia* collected from five ecoregions in the Atlantic Forest biome.

	ES	SM	ITA	MSP
ES				
SM	0.316			
ITA	0.322	0.164		
MSP	0.443	0.385	0.371	
DOU	0.427	0.430	0.451	0.369

ES, São Mateus ES; SM, Santa Maria RS; ITA, Itaparica BA; MSP, Morro de São Paulo BA; DOU, Dourados MS.

**Figure 2:**
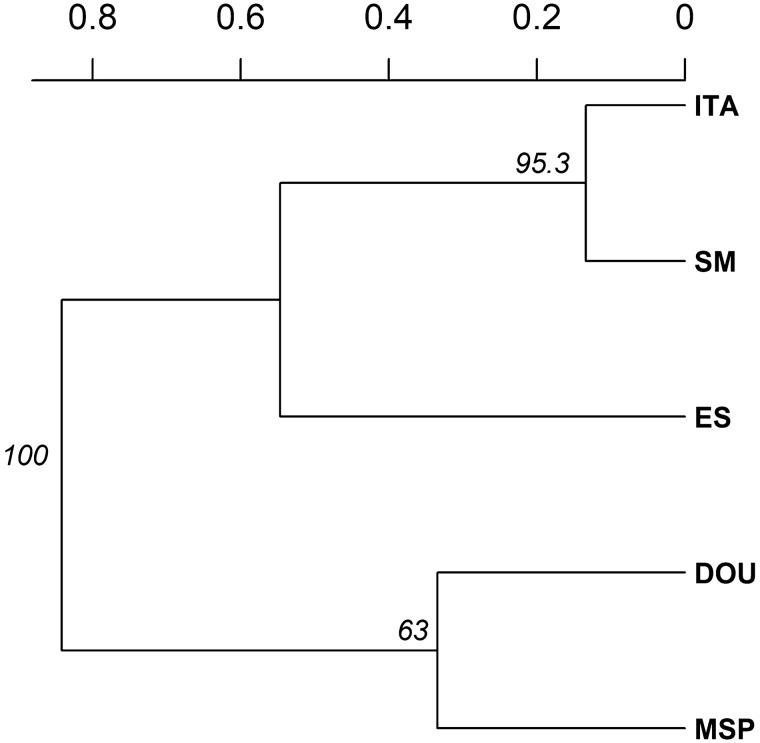
UPGMA based on Nei distance of seven microsatellite loci. The dendrogram represents genetic similarity among native populations of *S. terebinthifolia*. *ES, São Mateus-ES; SM, Santa Maria-RS; ITA, Vera Cruz-BA; MSP, Morro de São Paulo-BA; DOU, Dourados-MS.

UPGMA analysis also confirmed greater similarity between the SM and ITA populations, and revealed considerable distance between ITA and MSP, despite them being geographically close together in the same type of ecoregion (Fig. 2).

**Figure 3:**
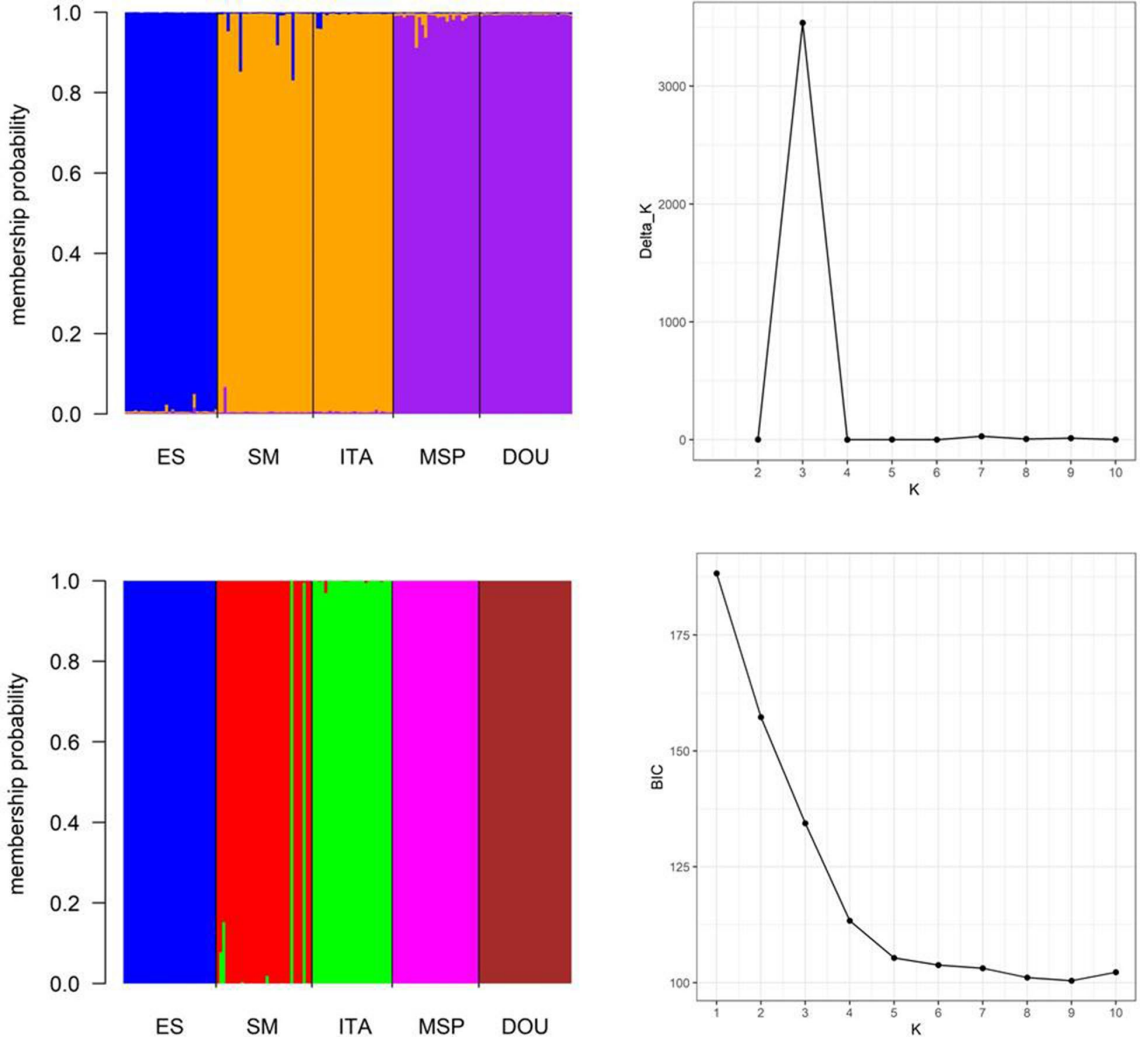
(**A**) results of a Bayesian analysis (STRUCTURE) of samples of *S. terebinthifolia* and estimates of the number of K groups based on the Delta K statistic of Evanno et al.^38^; (**B**) results of a DAPC analysis and the number of clusters as a function of the BIC values. Both results were based on the analysis of 145 samples using seven microsatellite loci, as indicated on the x-axis. Each column represents a different individual, and the colours represent the probability coefficient of each individual for each genetic cluster. *ES, São Mateus-ES; SM, Santa Maria-RS; ITA, Vera Cruz-BA; MSP, Morro de São Paulo-BA; DOU, Dourados-MS.

The STRUCTURE analysis and the Evanno method indicated that K = 3 was the most likely number of populations and grouped SM and ITA together and MSP and DOU together. In contrast, the DAPC analysis grouped individuals into their five respective populations with a few individuals in the SM population assigned to ITA (Fig. 3).

CG analysis detected 38 compounds, four of which were at relatively high concentration and found in all five populations including α-pinene, sabinene, β-pinene, limonene. A number of compounds were not observed in all five populations including ζ-salvene (only in MSP, SM and DOU), β-ocimene, evadone (only in ITA and MSP), aromadendrene, d-germacrene, d-davanone (only in MSP), carotol (only in ITA and SM) and eremoligenol (only in ES) (Table 6).

**Table 6:** Comparison of chemical profiles of the essential oil from fresh berries of *S. terebinthifolia* collected in five native populations from the Atlantic Forest Biome.

Compounds	RI	ITA (%)	ES (%)		MSP (%)	SM (%)	DOU (%)
cis-Salvene	856	–	–		1.96	4.34	4.70
α-Pinene	939	22.34	9.76		11.43	13.42	14.90
α-Fenchene	953	3.85	4.08		4.32	4.23	4.95
Sabinene	976	6.08	7.45		9.01	7.89	8.89
β-Pinene	980	9.56	14.03		8.63	8.52	12.99
Myrcene	991	0.36	0.18		0.25	0.19	0.11
α-Phellandrene	1009	0.37	0.17		0.28	0.46	0.19
Δ3-Carene	1011	0.28	–		0.29	0.15	0.17
Sylvestrene	1028	0.32	–		0.25	0.21	0.12
Limonene	1029	16.22	17.01		14.01	19.45	10.99
1,8-Cineole	1031	1.87	2.29		–	1.91	2.15
β-Phellandrene	1031	0.31	0.19		0.31	0.38	0.10
β-Ocimene	1037	1.44	1.42		3.37	1.34	1.26
γ-Terpinene	1060	–	1.69		2.55	1.61	1.73
Terpinolene	1088	0.25	0.16		0.56	0.27	0.10
Terpinen-4-ol	1176	1.22	1.64		1.38	–	1.51
α-Terpineol	1189	0.28	0.16		0.76	0.34	0.11
neo-iso-Verbanol	1189	–	2.22		2.54	2.52	2.71
ζ-Patchenol	1319	3.02	3.30		2.21	3.27	3.46
δ-Elemene	1339	0.21	0.12		0.51	0.30	0.12
Evodone	1340	1.34	–		1.04	–	–
α-Cubebene	1351	0.63	–		0.62	0.31	0.11
Isoledene	1373	0.20	–		0.51	0.21	0.10
Longicyclene	1375	2.46	1.91		1.95	1.95	1.03
α-Copaene	1377	6.34	7.35		9.03	9.09	10.15
β-Elemene	1391	0.29	0.12		0.11	0.38	0.13
Longifolene	1402	0.31	–		0.54	0.41	0.18
α-Funebrene	1403	2.57	2.05		3.05	2.19	3.33
Z-Caryophyllene	1404	0.27	0.11		0.59	0.42	0.15
Aromadendrene	1465	1.63	4.03		1.63	1.58	2.78
d-Germacrene	1485	3.24	8.82		6.86	4.80	3.67
Bicyclogermacrene	1500	3.44	1.33		3.38	1.37	1.32
δ-Cadinene	1514	1.89	1.34		1.79	1.94	2.13
Sphatulenol	1578	2.78	3.03		2.98	2.97	3.14
Globulol	1585	2.56	2.02		–	–	–
d-Davanone	1588	–	–		1.17	–	–
Carotol	1595	2.02	–		–	1.05	–
Eremoligenol	1631	–	1.65		–	–	–

RI, Retention index calculated; ITA, Itaparica (sandbank); ES, São Mateus (dense ombrophilous forest); MSP, Morro de São Paulo (sandbank); SM, Santa Maria (deciduous seasonal forest) and DOU, Dourados, MS (savanna). For those cells represented by “–” means the compound was not detected in that population.

**Figure 4:**
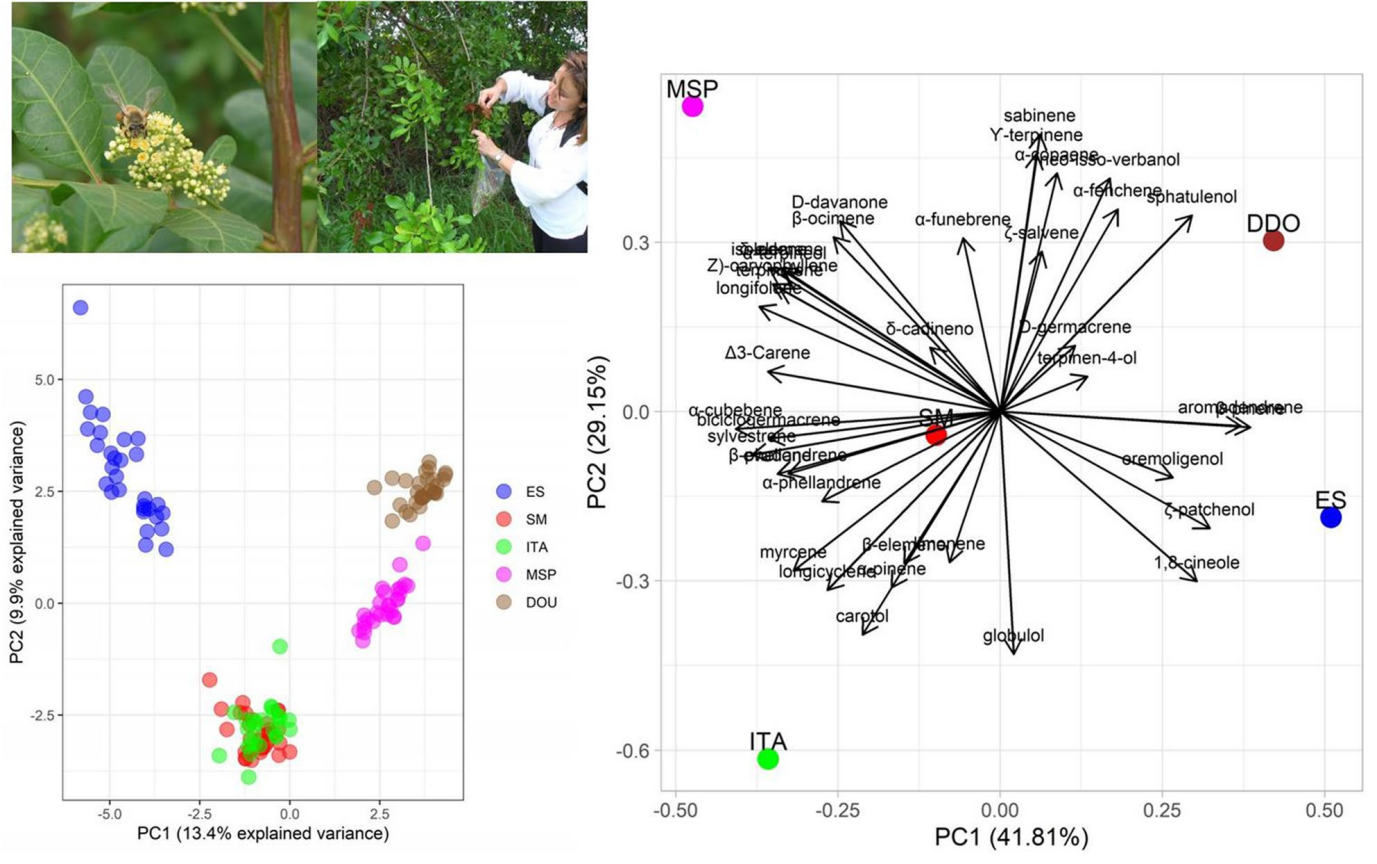
Cluster analysis based on PCA of genotypes and chemodiversity of native populations of *Schinus terebinthifolia* Raddi from five ecoregions in the Brazilian Atlantic Forest. ES, São Mateus-ES (dense ombrophilous forest); SM, Santa Maria-RS (deciduous seasonal forest); ITA, Itaparica-BA (sandbanks); MSP, Morro de São Paulo-BA (sandbanks); DOU, Dourados-MS (savanna).

Principal Components Analysis (PCA) plots were performed using genotypes and chemical compound data. Clusters found for genotypes reinforced the patterns of previous genetic structure analysis. ES individuals group in a separate cluster, while ITA and SM individuals cluster together. MSP and DOU individuals form two clusters that are close to each other and separate from the other populations. However, when the populations were grouped by their chemodiversity, the PCA revealed that each population has a unique chemical profile (Fig. 4).

## Discussion

As expected for an outcrossing species, the inbreeding coefficients (F_IS_) were low and genetic diversity was high in most of the sampled populations with the exception of DOU (Cerrado—Brazilian Savanna). The excess of heterozygotes and low allelic richness suggest this population has experienced a recent bottleneck^45,46^. This population is located in an area recently classified as an ‘Ecological Transition Zone’ due to anthropic pressures (deforestation, fire regime, settlement, etc.) which may be responsible for the bottleneck. Similar bottlenecks in Brazilian peppertree populations have been detected in other fragmented areas in Brazil and highlight the negative effect that anthropogenic factors can have on this species in its native range. The ES population was distinct in all clustering analyses and had the highest genetic diversity compared to the other sites. The ES population is located in an important conservation area (dense ombrophilous forest) which may have protected this population from genetic erosion. The presence of important species for phytomedicine such as Brazilian peppertree in these areas further supports the conservation of those forests.

This assumption can also be supported by the values of an observed heterozygosity higher than the expected (*He* 0.58 and 0.46, respectively). When a population experiences a reduction of its effective size, it generally develops a heterozygosity excess which may persist only for a certain number of generations until a new equilibrium is established^42^. Moreover, DOU showed the lowest rates for allelic richness (2.43). Reinforcing the hypothesis of bottleneck since the loss of the average number of alleles per locus is also related to genetic drift typical in populations that have their effective size dramatically reduced^43^.

To understand the genetic diversity of Brazilian peppertree it’s important to consider the high ecoplasticity and invasive behavior of the species. The populations analyzed in this work are originally from five different ecogeographic regions with particular characteristics (Table 1). Nevertheless, all five populations showed high genetic diversity (Table 4), especially when taking into account the rates from previous studies^21,44^, reinforced by a high differentiation index among populations (37.72%) and it proves that neither the time nor human pressure were enough to disrupt their distribution patterns—typical in invasive species. Possibly due to the high spatial density and efficient mechanisms for seed dispersal which allowed an intense gene exchange and consequent genotypic recombination, responsible to increase its evolutionary potential and adaptability.

Structure analysis (Fig. 3) showed us three clusters based on genetic similarity (SM + ITA; DOU + MSP and ES). Interestingly the clusters have no correlation with the distances where samples were collected, confirming what Ruas et al.^44^ had inferred about the species distribution patterns do not fit within the isolation by distance patterns. Pinto et al.^5^ have analyzed haplotypes distribution of Brazilian peppertree considering different biomes, and the authors suggested a cluster of 3 haplotypes based on the intravarietal polymorphisms. Nevertheless, in our study, population from ES showed the highest distance instead of DOU, while SM and ITA corroborate by clustering together. ES also presented the higher allelic richness (4.98), coincidentally this population occurs in the ecogeographic region of greater preservation compared to the others (dense ombrophilous forest).

The patterns of genetic variation across a species range can provide information about the processes of distribution and help us to understand how ecology, evolution and geography intersect^45^. We believe the similarities found between populations from SM and ITA may be correlated to the same colonization event and a reasonable explanation for divergence of the other three genotypes may be due to environmental barriers—DOU occurs in a fragmental zone; MSP in an isolated island and ES surrounded by the dense forest. Thus, an isolation by environment (IBE) could be expected under the action of natural selection and/or environmentally influenced mating or migration^46,47^. Furthermore, Williams et al.^21^ described the existence of well-structured populations of Brazilian peppertree in their native habitat. This phenomenon becomes evident in pairwise study when greater differentiation rates are obtained by DOU and MSP, respectively, two populations established in a narrow area and environmentally isolated^48,49^.

The dendrogram based on Nei distance (Fig. 2) confirms the occurrence of clusters, but if we consider the values on pairwise estimation (Table 5) the similarity between DOU and MSP is not relevant enough to confirm they are the same lineage. Still, it is necessary further analysis in comparison with other spatially intermediate populations to better understand migration patterns and distribution of the species.

The analysis of GC–MS of the essential oil of the fruits detected 38 different compounds (major and minor), some of which were not present in all five populations. The PCA revealed that each population had a unique chemical profile. Secondary metabolites are mostly related to environmental factors including soil properties, solar radiation, temperature, altitude, humidity and harvest time^22,23^, it would be unexpected some clusters formation. Consistent with this expectation, our sampled populations, with the exception of ITA and MSP, were in different ecosystems and had unique chemical profiles. ITA and MSP are located geographically close together in the same pedo-climatic conditions and yet they had the greatest divergences for chemical composition between the populations. This suggests that ecosystem designations are not capturing all of the relevant variables that could result in chemical profile differences. For instance, differences in predators, disease, or pollinators could also influence secondary metabolite synthesis. Future studies will need to take into consideration a wider variety of factors to determine which are important for differences in chemodiversity of populations.

From a pharmacological perspective, it is important to consider every factor that might influence the bioactivity of a phytomedicine. Understanding how environmental factors affect the chemodiversity of Brazilian peppertree will make it possible to develop elite lines for industry. As more studies are conducted across a wide variety of systems, we may be able to predict how different factors will influence patterns of chemodiversity.

## Data Availability

The datasets generated during and/or analysed during the current study are available from the corresponding author on reasonable request.
